# Flavonol-rich dark cocoa significantly decreases plasma endothelin-1 and improves cognition in urban children

**DOI:** 10.3389/fphar.2013.00104

**Published:** 2013-08-22

**Authors:** Lilian Calderón-Garcidueñas, Antonieta Mora-Tiscareño, Maricela Franco-Lira, Janet V. Cross, Randall Engle, Mariana Aragón-Flores, Gilberto Gómez-Garza, Valerie Jewells, Lin Weili, Humberto Medina-Cortina, Edelmira Solorio, Chih-kai Chao, Hongtu Zhu, Partha S. Mukherjee, Lara Ferreira-Azevedo, Ricardo Torres-Jardón, Amedeo D'Angiulli

**Affiliations:** ^1^Biomedical Sciences, The Center for Structural and Functional Neurosciences, The University of MontanaMissoula, MT, USA; ^2^Hospital Central Militar, Secretaría de la Defensa NacionalMexico City, Mexico; ^3^Instituto Nacional de PediatriaMexico City, Mexico; ^4^Department of Pathology, School of Medicine, University of VirginiaCharlottesville, VA, USA; ^5^Director Center for Advanced Brain Imaging, School of Psychology, Georgia TechAtlanta, GA, USA; ^6^Radiology Department, University of North Carolina Medical SchoolChapel Hill, NC, USA; ^7^Department of Radiology and Biomedical Research Imaging Center, University of North Carolina at Chapel HillChapel Hill, NC, USA; ^8^Biostatistics, University of North Carolina Gillings School of Global Public HealthChapel Hill, NC, USA; ^9^Mathematics Department, Boise State UniversityBoise, ID, USA; ^10^CAPES Foundation, Ministry of Education of BrazilBrasilia, Brazil; ^11^Centro de Ciencias de la Atmósfera, Universidad Nacional Autónoma de MéxicoMexico City, Mexico; ^12^Department of Neuroscience, Carleton UniversityOttawa, ON, Canada

**Keywords:** air pollution, Alzheimer's and Parkinson's disease risk, cocoa, cognition, children

## Abstract

Air pollution exposures are linked to systemic inflammation, cardiovascular and respiratory morbidity and mortality, neuroinflammation and neuropathology in young urbanites. In particular, most Mexico City Metropolitan Area (MCMA) children exhibit subtle cognitive deficits, and neuropathology studies show 40% of them exhibiting frontal tau hyperphosphorylation and 51% amyloid-β diffuse plaques (compared to 0% in low pollution control children). We assessed whether a short cocoa intervention can be effective in decreasing plasma endothelin 1 (ET-1) and/or inflammatory mediators in MCMA children. Thirty gram of dark cocoa with 680 mg of total flavonols were given daily for 10.11 ± 3.4 days (range 9–24 days) to 18 children (10.55 years, *SD* = 1.45; 11F/7M). Key metabolite ratios in frontal white matter and in hippocampus pre and during cocoa intervention were quantified by magnetic resonance spectroscopy. ET-1 significantly decreased after cocoa treatment (*p* = 0.0002). Fifteen children (83%) showed a marginally significant *individual* improvement in one or both of the applied simple short memory tasks. Endothelial dysfunction is a key feature of exposure to particulate matter (PM) and decreased endothelin-1 bioavailability is likely useful for brain function in the context of air pollution. Our findings suggest that cocoa interventions may be critical for early implementation of neuroprotection of highly exposed urban children. Multi-domain nutraceutical interventions could limit the risk for endothelial dysfunction, cerebral hypoperfusion, neuroinflammation, cognitive deficits, structural volumetric detrimental brain effects, and the early development of the neuropathological hallmarks of Alzheimer's and Parkinson's diseases.

## Introduction

Increasing evidence links neuroinflammation to neurodegenerative disease, particularly Alzheimer's disease (AD) (Santos et al., 2012a,b; Yan et al., 2013b; Sochocka et al., 2013). Air pollution exposures have been linked to neuroinflammation and neuropathology in young urbanites: 40% of highly exposed children and young adults exhibit frontal tau hyperphosphorylation with pre-tangle material and 51% have amyloid-β diffuse plaques as compared to 0% in low pollution controls (Calderón-Garcidueñas et al., 2012a, 2013). Clinically healthy Mexico City Metropolitan area (MCMA) children exhibit MRI prefrontal white matter hyperintensities (WMH) and significant selective impairment in attention, short term memory and learning ability in the absence of known risk factors for cognitive and neurological deficits (Calderón-Garcidueñas et al., 2008a).

Systemic inflammation and increased concentrations of potent vasoconstrictors (i.e., endothelin-1, ET-1) are key features of exposure in MCMA children, as they correlate with cumulative exposures to fine particulate matter (PM) and outdoor exposure hours, and are a reflection of the sustained inflammation of the upper and lower respiratory tracts and endothelial dysfunction (Calderón-Garcidueñas et al., 2003, 2007, 2008b). Air quality in MCMA stands among the worst in the world (Molina et al., 2007, 2010). Children are exposed all year long to a significant burden of air pollutants, including concentrations above the current US standards for ozone, and fine PM <2.5 μm in diameter (PM_2.5_) (Bravo-Alvarez and Torres-Jardón, 2002).

Given that neuroinflammation and endothelial dysfunction are critical for a proper brain growth and development, and based on our previous work on rodents showing that in MCMA exposed mice, dorsal vagal complex inflammation is mitigated by dark chocolate (Villarreal-Calderon et al., 2010), we explored the effects of a short cocoa intervention in MCMA clinically healthy children. There were two goals for this pilot study: (1) To quantify the responses of a selected group of cytokines, chemokines and ET-1, along with cognitive responses and brain MRS ratios in prefrontal white matter and hippocampus prior to and within 4 h of the last cocoa intervention day, and (2) To explore the children's acceptance of a daily morning administration of cocoa. Our guiding framework was that flavonols improve endothelial dysfunction and cognition parameters. Accordingly, we hypothesized that a short cocoa intervention could decrease the concentrations of a key marker of endothelial dysfunction ET-1 and consequently have an impact on critical brain metabolite ratios. Furthermore, as a byproduct of the coexistence between decreased ET-1 and positive changes in brain metabolites, MCMA children may show higher cognitive performance after the shortintervention.

## Procedure

### Study area

MCMA is the largest urban center in North America and a good example of extreme urban growth and environmental pollution (Bravo-Alvarez and Torres-Jardón, 2002; Molina et al., 2007, 2010). The metropolitan area of over 2000 square kilometers is home to 20 million inhabitants including 8 million children. The energy demand of this population, over 40,000 industries and 4 million vehicles consume more than 40 million liters of petroleum fuels per day producing an annual emission of ~2.6 tons of pollutants including coarse and fine PM, gaseous pollutants, polycyclic aromatic hydrocarbons, and lipopolysaccharides (Bravo-Alvarez and Torres-Jardón, 2002; Molina et al., 2007). The MCMA is located in the southwestern portion of an elevated basin 2240 m above sea level that is surrounded on three sides by mountain ridges at 19°N 99°W. The high altitude and tropical insolation of the basin facilitate ozone production all year and contribute to the formation of secondary PM (Bravo-Alvarez and Torres-Jardón, 2002). Although substantial reductions in the concentrations of some criteria pollutants (i.e., lead, CO, and SO_2_) have been achieved during the past 15 years, MCMA residents remain exposed to concentrations of airborne pollutants exceeding US ambient air quality standards for PM and ozone.

For this work, we will focus on ozone and PM broadly defined by the diameter of the aerodynamic particles, and classified into coarse particles (<10 μm; PM_10_), fine particles (<2.5 μm; PM_2.5_) and ultrafine particles (<100 nm; UFPM). Fine and ultrafine PM are of particular interest given their capability to reach the brain (Block and Calderón-Garcidueñas, 2009). In this massive exposure chamber, 8 million children and teens are enduring the consequences of their involuntary exposure to the polluted air.

### Participants and cocoa intervention

The research was approved by the Institutional Review Boards of the involved hospitals in Mexico City, children gave active assent and their parents gave written informed consent to participation. This work includes data from 18 MCMA children (*Mean age* = 10.55 years, *SD* = 1.45; 11F and 7M) carefully selected to represent comparable populations recruited for a larger longitudinal cohort research program. Clinical inclusion criteria for all children were: negative smoking history and environmental tobacco exposure, lifelong residency in MCMA, residency within 5 miles of the city monitoring stations, full term birth, and unremarkable clinical histories, including no hearing or visual impairments. Children were matched by age, socioeconomic status (SES) and had similar BMI. In addition to the general inclusion criteria, the specific criterion for the selection included a negative history of food allergies, including chocolate allergies or lactose intolerance. The cocoa powder selected (D'Vicar, Coyoacán, Mexico City) contained 680 mg of total flavonols per 30 g and it was administered for 10.11 ± 3.4 days (minimum 9 days, maximum 24 days). A cold milk shake or hot milk containing premeasured 30 g of cocoa with sugar *ad-libitum* was part of the participants' morning breakfast. There were no changes in their normal diet with the exception that the child consumed no chocolate, high flavonol foods and nutritional complements in the 15 days prior to the beginning of the intervention, and had no high flavonol products during the cocoa intervention period.

### Pediatric examination

Children included in this study have been followed by our pediatricians for a minimum of 2 years, had initial complete clinical histories and physical examinations, and underwent two annual pediatrician check-up visits. At the time of the intervention, all included children were clinically healthy and similarly actively engaged in outdoor activities (*range*: 3.2–4.9 h daily).

### Peripheral blood analysis

Blood samples were collected for a complete blood count (CBC) with differential and Human 41-Plex Primary Cytokine/Chemokine Panel (Multiplexing LASER Bead Technology, Eve Technologies, Calgary, Alberta, Canada T3A OZ9): EGF, Eotaxin, FGF-2, Flt-3L, Fractalkine, G-CSF, GM-CSF, GRO, IFNα 2, IFN γ, IL-1α, IL-1β, IL-1ra, IL-2, IL-3, IL-4, IL-5, IL-6, IL-7, IL-8, IL-9, IL-10, IL-12 (p40), IL-12 (p70), IL-13, IL-15, IL-17a, IP-10, MCP-1, MCP-3, MDC, MIP-1α, MIP-1β, PDGF-AA, PDGF-AB/BB, RANTES, sCD40L, TGFα, TNFα, TNFβ, VEGF.IL-1α, IL-1β, IL-1ra, IL-2, IL-3, IL-4, IL-5, IL-6, IL-7, IL-7, IL-8, IL-9, IL-10, IL-12 (p40), IL-12 (p70), IL-13, IL-15, IL-17, IP-10, MCP-1, MCP-3, MDC, MIP-1α, MIP-1β, PDGF-AA, PDGF-AB/BB, RANTES, sCD40k, sIL-2Rα, TGFα, TNFα, TNFβ, VEGF, IFN α 2, IFN γ, G-CSF and GM-CSF. ELISAS from R&D were selected for ET-1 (DET-100, sensitivity: 0.207 pg/mL).

### Cognitive profiles

Children in this study had cognitive profiles measured using the subscales of the Wechsler Intelligence Scale for Children-Revised (WISC-R) (Information, Similarities, Arithmetic, Vocabulary, Comprehension, Digit Span, Picture Arrangement, Block Design, Object Assembly, Coding, and Mazes) within 3 months of their participation in this study. Children had a Full Scale IQ total of 110.6 ± 10.9 *SD*, Performance IQ of 108.8 ± 12.3 *SD* and a Verbal IQ of 109.6 ± 12.7 *SD*. Within 24 h prior to the beginning of the cocoa intervention and within 4 h of the last intervention day, children underwent two simple span tasks aim to measure short term memory, a subcomponent of working memory (Engle and Oransky, 1999; Engle and Kane, 2004; Shipstead et al., 2012). The Spanish version of the laboratory cognitive test system designed by Engle (2002, 2010) was used. Such system was designed to measure running memory span and to predict fluid intelligence by assessing both verbal and non-verbal information. Consequently, this system consists of two computer-based tasks. In the *Running Object Span (Images)* children see a list of 3–11 simple and distinguishable line drawings of concrete objects presented one at a time at a rate of 1 per s. Children see 3–11 objects and recall the last 3–8 of them using the touch screen monitor. In the *Recall Letter Span (Letters)*, the children see a list of 3–11 letters and touch the square corresponding to each letter in the list in turn. Performance on both tests was measured before and after the cocoa intervention. The administration of the tests was run by a trained experienced pediatric psychologist; each testlasted ~10 min.

### Brain magnetic spectroscopy imaging (MRS)

**Figure 1:**
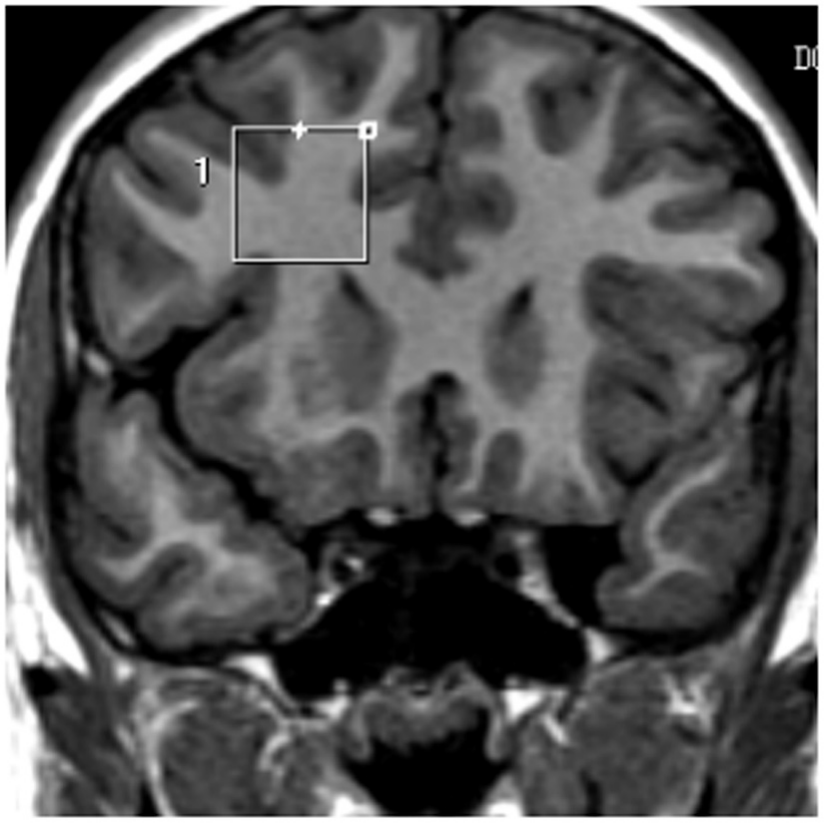
**Proton spectra was obtained from a voxel 2 × 2 × 2 cm prescribed from a coronal plane.** Four MRS VOI prescriptions were made for each child: two on the prefrontal white matter (right and left hemisphere) placing the VOI just ahead from the frontal horns of the lateral ventricles.

**Figure 2:**
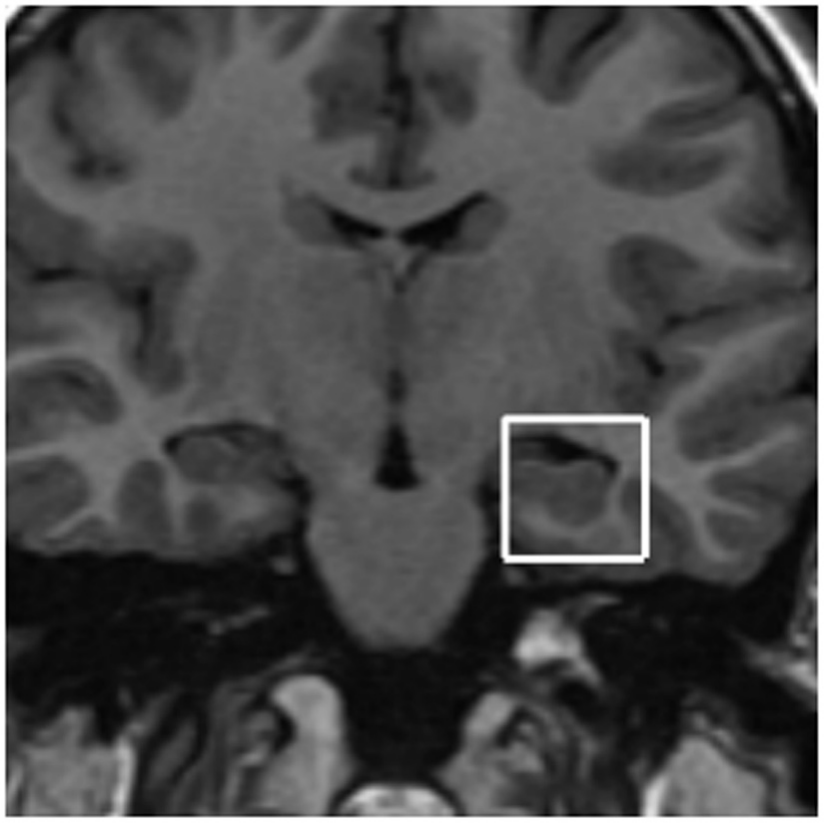
**Proton spectra was obtained from a voxel 2 × 2 × 2 cm prescribed from a coronal plane.** Four MRS VOI prescriptions were made for each child: two on medial hippocampal regions (right and left hemisphere each).

All 18 children underwent a brain MRS 24 h prior to the start of the cocoa intervention and within 4 h of the last morning intervention. Children were not sedated. The 3D MRS for all subjects was acquired on a 1.5 Tesla 5T Signa Excite HD MR (General Electric, Milwaukee WI, USA) with an 8 Channel Phased Array head coil. The goal of the selected acquisition protocol was to allow for brain metabolite assessment bilaterally in two anatomical regions: hippocampus and prefrontal white matter. Proton spectra was obtained from a voxel 2 × 2 × 2 cm prescribed from a coronal plane. Two sets of images where acquired on orthogonal planes: sagittal T1 flair (TR: 2238 ms; TE: 27.2 ms; TI:750 ms; NEX: 2; FOV 21 × 21; 5 mm thickness; 1 mm spacing), and coronal T1 flair (Tr: 1538 ms; TE: 23.7 ms; TI: 708 ms; NEX: 2; FOV 22 × 17.6; 3.0 mm thickness; 0.0 spacing). ^1^H MRS was obtained from single voxel of interest (VOI) of 8 cm^3^ (2 × 2 × 2 cm), with the Probe-P sequence, TR: 1500 ms; TE 35 ms, with automatic shimming and water supression, time of scan 2′12″. Four MRS VOI prescriptions were made for each child: two on the prefrontal white matter (right and left hemisphere) placing the VOI just ahead from the frontal horns of the lateral ventricles (Figure 1); two on medial hippocampal regions (right and left hemisphere each) trying to include the entire hippocampus and medial temporal cortex (Figure 2). In all subjects, the T1-weighted image covers the target areas including the left and right prefrontal cortex, and the hippocampus. For the purposes of this study we used the following metabolites ratio data: N-acetylaspartate/creatine *(*NAA/Cr), choline/creatine (Cho/Cr), and myoinositol/creatine (mI/Cr). For the quantification of the absolute metabolite concentrations, we used the user-independent frequency domain-fitting program LC Model version 6.1, with an automatic eddy correction and water suppression.

### Data analysis

First, we calculated the summary statistics (mean ± standard deviation) of all relevant variables including age, weight, height, BMI, cocoa intervention time, cumulative air pollutants at the time of the experiment in MCMA, blood laboratory results, inflammatory mediators and ET-1 results, and target brain metabolites before and after cocoa intervention. Next, we carried out statistical tests to investigate whether the differences of blood laboratory results, inflammatory mediator and ET-1 measurements in the two groups i.e., before and after cocoa intervention, were significant. We performed Wilcoxon rank sum test (WRST) and establish significance if the *p*-value of WRST was < 0.05. Next, we considered the post and pre-cocoa differences in blood count variables, cytokines and the MRS ratio variables as “responses” and the air pollutant variables as “predictors.” We carried out the regression analysis individually for each pair of such differences and air pollutants. While performing such regression analyses, we calculated multiple *R*^2^-values and the *p*-values for the significance of the linear regressions. We concluded that a difference has significant linear association with an air pollutant if the corresponding *p*-value is < 0.05. For the cognitive tests, we calculated the differences between pre-cocoa and post-cocoa scores for both Images and Letters tasks and tested through binomial test whether *improvement* defined as the direction of higher scores at retest in one or both tasks occurred in more individuals than those expected by chance (assuming *p* = 0.05). In addition, we performed WRST as well to assess group performance differences between test and retest in the two tasks; lack of group differences and smaller discrepant effect sizes between the individual and group tests may support the hypothesis that individual improvements, although modest, were likely not due to practice effects. All the statistical analyses described above were performed on statistical software “*R*” (http://www.r-project.org/).

## Results

### Air quality data

**Figure 3:**
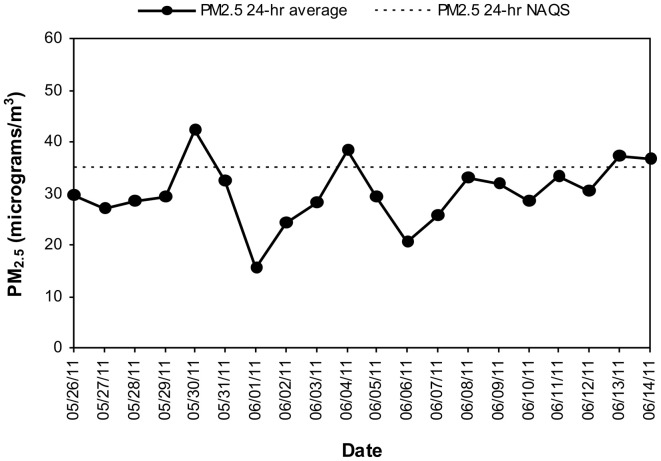
**Time series of 24-h average PM_2.5_ in the MCMA children's residency area from May 26 to June 14, 2011.** The dashed line shows the US EPA NAAQS value of 24-h average PM_2.5_ concentration which stands for the 98th percentile value of all days in the year.

**Figure 4:**
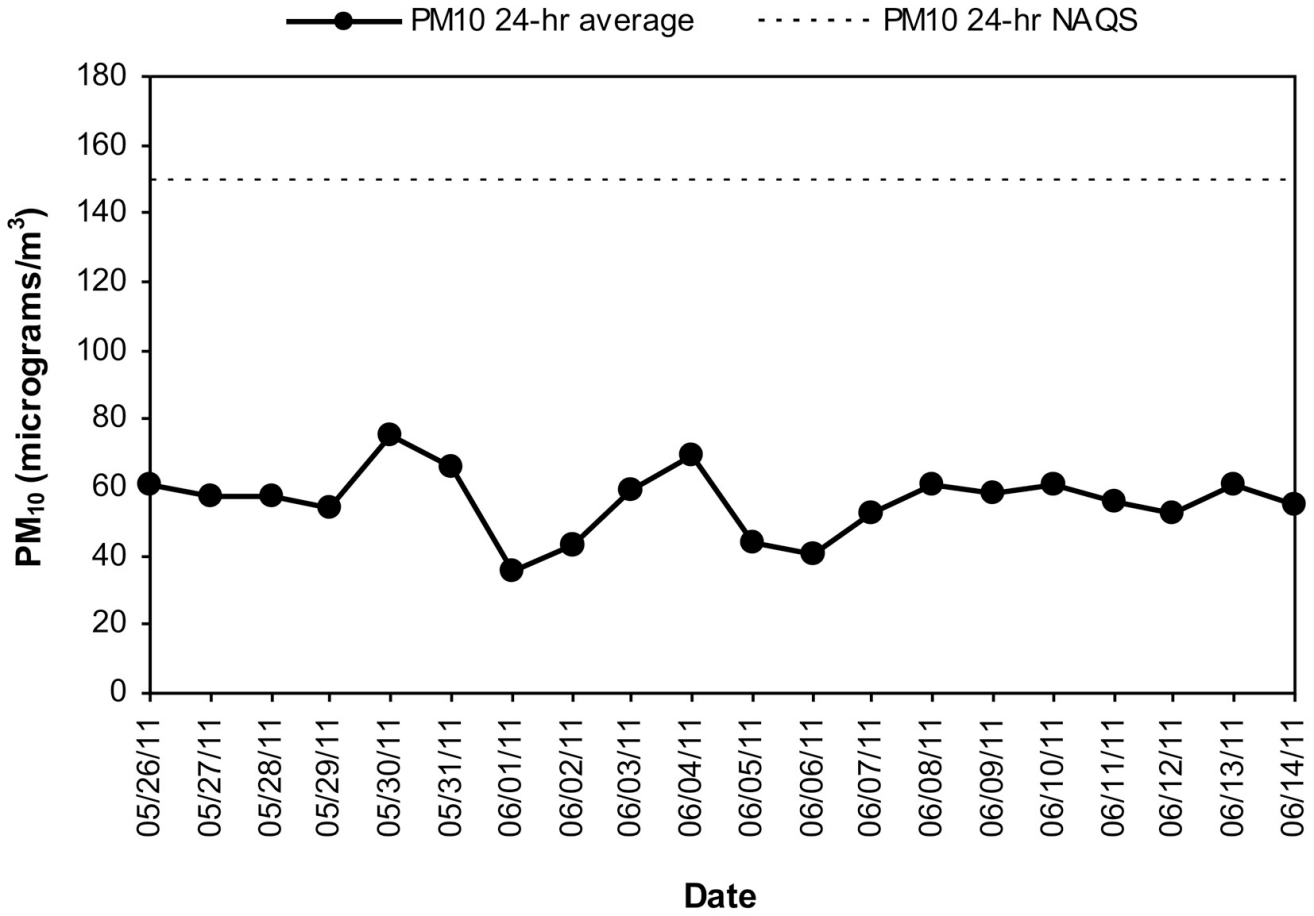
**Time series of 24-h average PM_10_ in the MCMA children's residency area from May 26 to June 14, 2011.** The dashed line shows the US EPA NAAQS value for PM_10_ 24-h average concentration.

**Figure 5:**
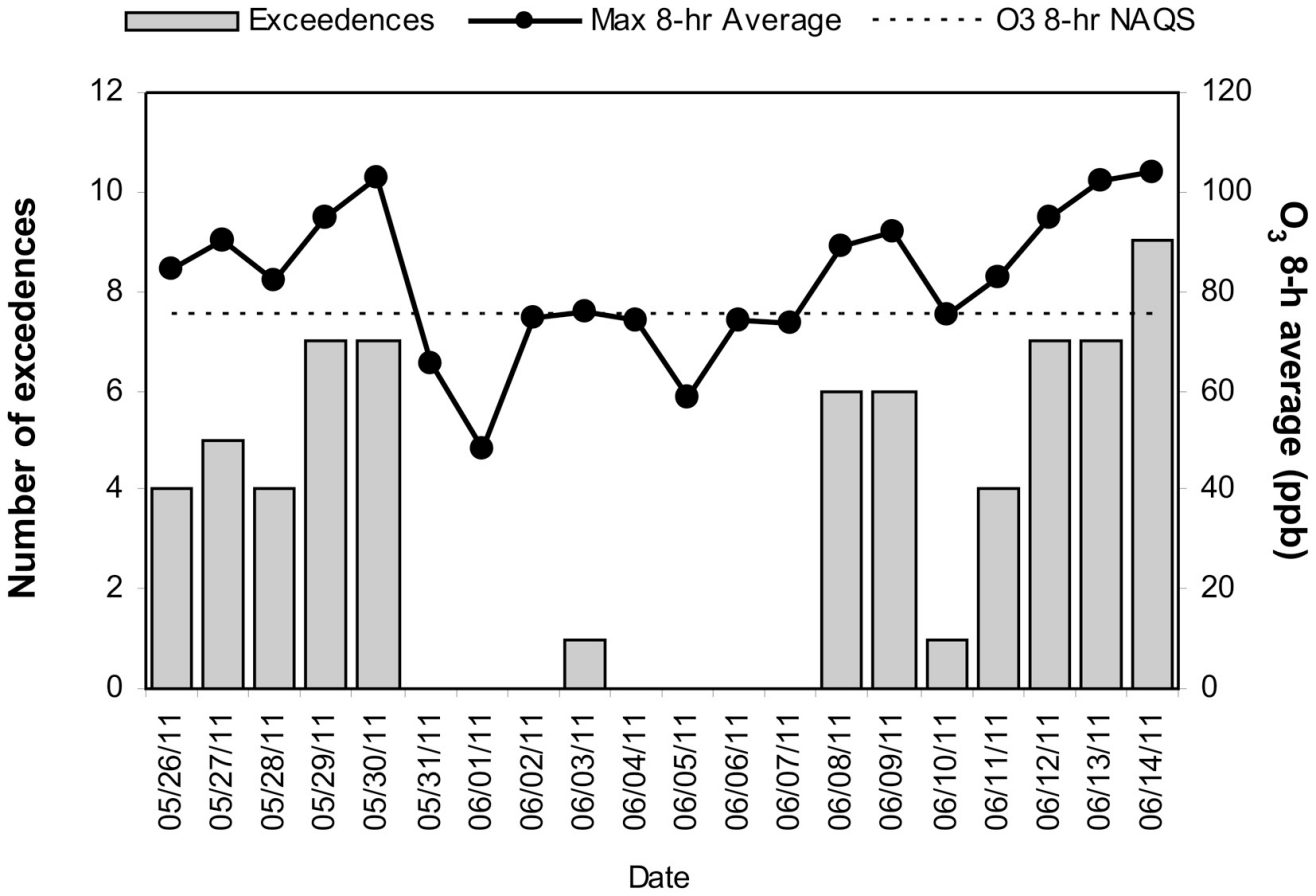
**Time series of the daily number of exceedences to the US EPA NAAQS value for the highest daily ozone 8-h average concentration and the respective daily maximum ozone 8-h average concentration in the children's residency area from May 26 to June 14, 2011.** The dashed line shows the ozone 8-h NAQS value as a reference.

MCMA children in this study were exposed to significant concentrations of ozone (O_3_), and PM before and during the study period. Table 1 illustrates the cumulative concentrations 24, 48, 72 and 7 day pre-cocoa and at the last intervention day-of the main criteria pollutants affecting the region (ozone, PM_10_, PM_2.5_). The concentrations of these criteria pollutants obtained from a monitoring station less than 5 miles from the child residency and school location showed no statistical differences throughout the study period or in the week before the beginning of the intervention. Figure 3 shows the 24 h average concentrations for PM _2.5_ in the children's residency area for the study period. The 24-h average PM_2.5_ concentrations were mostly below the 24 h air quality standard of 35 μ g/m^3^, while PM_10_ concentrations were always below the 24 h standard of 150 μ g/m^3^ (Figure 4). Ozone 8 h mobile average on the other hand (Figure 5) went above the standard (0.075 ppm on an 8 h averaging time) 65% of the monitored period. All other criteria pollutants, including nitrogen dioxide, sulfur dioxide and lead were below the current EPA standards (data not shown). The climatic conditions in MC are relatively stable, thus, pollutant concentrations are consistent year after year. The annual arithmetic mean of PM_2.5_ average concentrations in the study area (March 2009–March 2011) was 24.6 μ g/m^3^ (US EPA PM_2.5_ annual arithmetic mean standard stands for 12 μ g/m^3^). It is not unusual to have PM_2.5_ 1 h average concentrations reached values as high as 90 μ g/m^3^ during the mid-morning school recess time. The high concentrations of the main criteria pollutants coincide with the time children play outdoors and/or stay in schools with broken windows and doors (Villarreal-Calderón et al., 2002).

**Table 1:** **Cumulative air pollutant results in Mexico City children pre and post-cocoa**.

	**Pre-cocoa**	**At the last cocoa intervention day**
PM2.5 24 h^*^	23.2 ± 3.3	23.9 ± 1.4
PM2.5 48 h^*^	49.8 ± 11.9	46 ± 0
PM 2.5 72 h^*^	77.1 ± 12.4	69.6 ± 0.48
PM2.5 7 days^*^	195 ± 10.05	171.6 ± 3.39
PM10 24 h^*^	53.3 ± 10.7	53.2 ± 2.4
PM10 48 h^*^	113.8 ± 31	103.6 ± 1.9
PM10 72 h^*^	174.4 ± 27.9	156.7 ± 0.48
PM10 7 days^*^	432.9 ± 25.3	381.3 ± 5.5
Ozone 24 h^§^	969.2 ± 13.3	1258 ± 0.9
Ozone 48 h^§^	2072 ± 314	2513 ± 44
Ozone 72 h^§^	3327 ± 493	3662 ± 109
Ozone 7 days^§^	8218 ± 266	8111 ± 110

The numerical values shown in the table represent means (± standard deviation).

* *μ g/m^3^*,

§ ppm (parts per million). The air pollutant values were taken from the closest monitoring station (<5 miles) to the children's residency and school.

### Anthropometric measurements, physical examination

Children included in this study had complete unremarkable clinical histories and physical exams, and were actively engaged in outdoor activities (*range*: 3.2–4.9 h daily). Their BMI's (*M* = 17.8, *SD* = 2.14) and height (*M* = 1.4 m, *SD* = 0.1) were within normal values for their age and gender.

### Laboratory findings

Peripheral blood results before and after an average of 10.11 ± 3.4 days with cocoa intervention are shown in Table 2. There was a trend for the reticulocytes to go down, and the total neutrophils and platelets to go up after cocoa treatment, but it did not reach statistical significance. All hematological results are within normal limits for Mexico City. Table 3 shows the results of the inflammatory mediators examined. Plasma endothelin-1 concentrations significantly decreased after the cocoa treatment (*p* = 0.0002). Significant post-cocoa intervention differences in total polymorphonuclear leukocytes (PMN), eosinophils and basophils were associated with PM_2.5_ and ozone concentrations (Table 4). Four hour post-cocoa concentrations of selected cytokines on the last intervention day (IL7, IP-10, PDGF-AA, G-CSF, IL1β, IL2) were significantly associated with cumulative PM_2.5_ and ozone concentrations (Table 5).

**Table 2:** **Blood laboratory results in Mexico City children pre and post-cocoa**.

	**Pre-cocoa**	**Post-cocoa**	***p*-value**
Hemoglobin (g/dL)	14.7 ± 0.6	14.6 ± 0.5	0.9
Hematocrit (%)	44 ± 1.9	43.11 ± 1.4	0.16
Reticulocytes (%)	1.8 ± 0.5	1.4 ± 0.5	0.06
WBC (×10^3^/dL)	5.9 ± 1.1	6.5 ± 1.3	0.12
Neutrophils (%)	47.3 ± 9.4	47.2 ± 13.7	0.87
Lymphocytes (%)	41.4 ± 7.9	41.1 ± 12.7	1
Monocytes (%)	6.7 ± 1.8	6.6 ± 1.9	0.83
Eosinophils (%)	3.0 ± 2.7	3.4 ± 2.8	0.28
Basophils (%)	0.47 ± 0.2	0.55 ± 0.2	0.21
Total neutrophils	2.9 ± 1.1	3.2 ± 1.4	0.73
Total lymphocytes	2.4 ± 0.3	2.6 ± 0.6	0.29
Total monocytes	0.4 ± 0.09	0.43 ± 0.13	0.57
Total eosinophils	0.2 ± 0.2	0.26 ± 0.2	0.67
Platelets ×10^9^/L	264.6 ± 35.2	280.4 ± 47.4	0.28
Glucose mg/dL	87.6 ± 5.1	88.6 ± 5.3	0.54
Cholesterol mg/dL	161.5 ± 9	156.7 ± 31.5	0.60
Triglycerides mg/dL	98.9 ± 45	118.7 ± 62.2	0.28
HDLD mg/dL	45.8 ± 9.9	44.9 ± 11.5	0.57
LDL mg/dL	95.9 ± 22	88.14 ± 26	0.38
VLDL mg/dL	19.6 ± 9.1	23.7 ± 12.4	0.28

The numerical values shown in the table represent means (± standard deviation).

**Table 3:** **Inflammatory mediator and ET-1 results pre and post-cocoa intervention**.

	**Pre-cocoa**	**Post-cocoa**	***p*-value**
Eotaxin	41 ± 27	44.2 ± 25	0.51
FGF-2	48.0 ± 38	54.7 ± 40.3	0.43
FLT-3	9.4 ± 8.4	9.3 ± 4.2	0.39
Fractalkine	76.8 ± 184	76.3 ± 175	0.12
G-CSF	5.1 ± 2.7	8.5 ± 9.6	0.35
GM-CSF	13.9 ± 10	16.4 ± 13	0.42
GRO	407 ± 240	472.6 ± 283	0.30
IFNα	5.4 ± 6.3	4.8 ± 5.2	0.93
IFNγ	9.5 ± 15.1	7.8 ± 12	0.81
IL1α	6.5 ± 9.2	6.2 ± 9.6	0.96
IL1β	3 ± 6.3	2.9 ± 4.3	0.55
IL1r	5.5 ± 9.6	5.3 ± 5.58	0.55
IL2	4.6 ± 11.2	4.7 ± 9.7	0.94
IL6	2.5 ± 3.7	3 ± 4.2	0.57
IL7	2.7 ± 3.2	2.5 ± 1.7	0.51
IL8	6.3 ± 6.2	7.9 ± 7.9	0.36
IL10	1.8 ± 1.2	2.1 ± 1.4	0.53
IL12p40	13.4 ± 20.5	11.8 ± 12.1	0.86
IL17	6.7 ± 16.4	6.9 ± 13.9	0.88
IP10	82.8 ± 25	104.4 ± 63	0.86
MCP-1	271.9 ± 170	345.2 ± 200	0.16
MCP-3	11.6 ± 13.9	11.9 ± 12.8	0.76
MDC	1754 ± 1456	2471 ± 2080	0.13
MIP-1α	37.4 ± 36.3	40.1 ± 35	0.92
MIP-1β	27.3 ± 40.2	29.1 ± 46	0.76
PDGF-AA	288 ± 223	291 ± 202	0.86
PDGF-AB	9267 ± 4535	10477 ± 4343	0.38
RANTES	1035 ± 490	1219 ± 585	0.35
CD40L	1592 ± 3255	1544 ± 2596	0.30
sIL-2rα	10.6 ± 13	9.8 ± 11.2	0.98
TGFα	4.9 ± 5.6	6.1 ± 7	0.64
TNFα	2.4 ± 2.5	2.7 ± 2.4	0.41
TNFβ	2.4 ± 3.3	2.3 ± 2.9	0.78
VEGF	23.3 ± 24.6	25.2 ± 22.8	0.64
ET-1	1.584 ± 0.35	0.9567 ± 0.4054	0.0002

The numerical values shown in the table represent means (± standard deviation). Concentrations are given in pg/ml.

**Table 4:** **Statistically significant post-cocoa intervention differences in complete Blood Count (CBC) variables associated with PM and ozone cumulative values**.

**Pollutant variables**	**Total PMN**	**Total eosinophils**	**Total basophils**	**Cholesterol**
	***R*^2^/*p***	***R*^2^/*p***	***R*^2^/*p***	***R*^2^/*p***
PM2.5 24 h		0.51/0.001		
PM2.5 48 h				
PM2.5 72 h				
PM2.5 7 days	0.31/0.02			
PM10 24 h		0.39/0.009		
PM10 48 h				
PM10 72 h				
PM10 7days				
Ozone 24 h		0.55/0.0009		
Ozone 48 h				
Ozone 72 h				
Ozone 7 days			0.64/0.0001	0.23/0.04

**Table 5:** **Statistically significant post-cocoa intervention differences in selected cytokines and chemokines variables associated with PM and ozone cumulative values**.

**Pollutant variables**	**IL-7**	**IP-10**	**PDGF-AA**	**G-CSF**	**IL1β**	**IL2**
	***R*^2^/*p***	***R*^2^/*p***	***R*^2^/*p***	***R*^2^/*p***	***R*^2^/*p***	***R*^2^/*p***
PM2.5 24 h		0.39/0.005				
PM2.5 48 h			0.22/0.04			
PM 2.5 72 h						
PM2.5 7 days	0.25/0.03					
PM10 24 h		0.37/0.006	0.24/0.03			
PM10 48 h						
PM10 72 h						
PM10 7days	0.24/0.03					
Ozone 24 h						
Ozone 48 h		0.28/0.02	0.24/0.03			
Ozone 72 h						
Ozone 7 days				0.6/ 0.0001	0.3/0.01	0.34/ 0.01

### Cognitive data

Fifteen children (83%) showed a marginally significant *individual* improvement more frequently in the retest in one or both of the applied simple short memory tasks (Binomial Test, *p* = 0.048, one-tailed). Mean scores for Letters was 15.39 (*SD* = 6.08) vs. 16.72 (*SD* = 7.22) and for Images 13.11 (*SD* = 5.41) vs. 13.11 (*SD* = 6.53) on pre and post-cocoa tests, respectively. However, there were no statistical differences in mean performance scores for the groups (*p* = 0.46 for Letters and *p* = 0.89 for Images) and the effects were much discrepant from the one observed for the binomial test. Therefore, most likely the improvement observed at the level of the individual cases was not due simply to practice otherwise we would have obtained significance or similar effect sizes.

### MRS data

Two pediatric radiologists carried out the T1 visual evaluation. Post-cocoa differences in the MRS ratio variables in the pre frontal right and left white matter were associated with cumulative PM_2.5_ and ozone concentrations (Table 6). No significant statistical differences were seen pre and post-cocoa with the targeted MRS ratios (NAA/Cr, Cho/Cr, mI/Cr, GABA/Cr, GLU/Cr, LAC/Cr, TAU/Cr, GUA/Cr) (all *p*-values >0.05, data not shown). Changes in the concentration of ET-1 after cocoa intake were not significantly related with MRS variables (all *p*-values >0.05).

**Table 6:** **Statistically significant post-cocoa intervention differences in selected frontal MRS ratios associated with PM and ozone cumulative values**.

**Pollutant variables**	**Frontal R Cho/Cr**		**Frontal L NAA/Cr**		**Frontal L mI/Cr**	
	***R*^2^**	***p*-value**	***R*^2^**	***p*-value**	**R^2^**	***p*-value**
PM2.5 24 h						
PM2.5 48 h			0.42	0.0214	0.50	0.0146
PM 2.5 72 h	0.45	0.032	0.40	0.0250	0.51	0.0125
PM2.5 7 days			0.42	0.0213	0.51	0.0126
PM10 24 h						
PM10 48 h			0.42	0.0212	0.51	0.013
PM10 72 h	0.47	0.028	0.39	0.0290	0.50	0.013
PM10 7 days			0.42	0.0216	0.51	0.0123
Ozone 24 h						
Ozone 48 h			0.40	0.0260	0.45	0.0226
Ozone 72 h			0.42	0.0213	0.51	0.0126
Ozone 7 days			0.42	0.0220	0.49	0.0158

## Discussion

A short high flavonol cocoa intervention significantly decreased plasma concentrations of endothelin-1 and improved cognition in highly exposed urban children. Endothelial dysfunction, defined as an imbalance between relaxing and contractile endothelial factors, plays a critical role in the pathogenesis of a wide variety of detrimental effects upon exposures to air pollutants, especially PM (Thomson et al., 2005, 2007; Calderón-Garcidueñas et al., 2007, 2008b; Tamagawa et al., 2008; Lund et al., 2009; Matsumoto et al., 2010; Cao et al., 2012; Krishnan et al., 2012; Veras et al., 2012; Miyata et al., 2013). Acute and chronic exposures to vehicular source pollutants and PM components have been associated with endothelial dysfunction and the development and progression of a wide range of cardiovascular diseases, activation of ET-1-ET (A) receptor pathways, persistent lung inflammation, up-regulation of cerebro-vascular ETA receptors via the Raf/ERK/MAPK pathway, and increases in plasma ET-1 and pulmonary artery pressure (Calderón-Garcidueñas et al., 2007; Tamagawa et al., 2008; Lund et al., 2009; Cao et al., 2011; Krishnan et al., 2012). Mice exposed to ambient urban air exhibit decreases in the lumen/wall ratios of lung arterioles on the early days of exposure compared with clean air controls (Matsumoto et al., 2010), while mice fetuses exposed to the same urban environment have extensive umbilical cord vessel structural changes with increased immunoreactivity of isoprostane and ET receptors, indicative of vascular dysfunction and oxidative stress (Veras et al., 2012). PM and ozone independently regulate the expression of lung, brain and pituitary endothelin system genes (Thomson et al., 2005, 2007). Mice exposed to the same polluted MCMA atmosphere as children, responded with significant decrease in both myocardial and brain inflammation after dark chocolate interventions (Villarreal-Calderon et al., 2010; Villarreal-Calderon et al., 2012).

Of critical importance for urban children is the recent work by D'Haeseller et al. establishing the reversibility of cerebral hypoperfusion mediated by endothelin 1 in multiple sclerosis (MS) patients (D'Haeseller et al., 2013). The source of ET-1 in MS is likely reactive astrocytes around MS plaques (D'Haeseller et al., 2013). MCMA children exhibit prefrontal white matter hyperintensities (WMH), extensive diffuse vascular lesions with breakdown of the blood-brain-barrier (BBB), and high plasma ET-1 that strongly correlated with daily outdoor hours, and 7-day cumulative levels of PM_2.5_ before ET-1 measurement (Calderón-Garcidueñas et al., 2007, 2008b, 2012a). These findings in highly exposed urban children suggest that inflamed brain and lungs (Calderón-Garcidueñas et al., 2007) could be key sources of ET-1 (Thomson et al., 2005, 2007) and as proposed by Taylor et al. focal brain vascular damage, hypoperfusion and hyperintense white matter lesions could alter neural connectivity (Taylor et al., 2013) and thus cognition.

The human brain is extremely sensitive to hypoperfusion, endothelial dysfunction, chronic intermittent hypoxia, and glucose dysmetabolism (Daulatzai, 2012). Hypoxia/hypoxemia underpins several pathological processes including neuroinflammation, oxidative stress, and a host of pathways leading to neurodegeneration (Daulatzai, 2012). Chronic cerebral hypoperfusion is included as a prodromal feature of aging-related dementias (Romanini et al., 2013) and subclinical ischemia impairs cognitive performance (Inoue et al., 2013).

Moreover, since the presence of WMH in older adults with cardiac failure showed a significant reduction of cerebral blood flow (CBF) in the middle cerebral artery (Alosco et al., 2013), measurement of CBF in exposed children ought to be included in future research protocols.

Mexico City children are also showing differential white and gray matter volumetric responses mediated by cytokines and chemokines involved in resolution of inflammation, immunoregulation, and tissue remodeling (Calderón-Garcidueñas et al., 2012b). This issue warrants further study in longer cocoa intervention protocols, since the cocoa's impact on cytokines and chemokines with significant immune modulatory effects and neuroprotection, i.e., G-CSF and CCL22 will be of key relevance for highly exposed children. Interestingly, in this study Granulocyte colony-stimulating factor (G-CSF) was significantly associated with ozone cumulative 7 *d*-values. This cytokine down-regulates the expression of pro-inflammatory cytokines and increases powerful anti-inflammatory ones like IL10 in a rodent model of experimental autoimmune neuritis, thus displaying a neuroprotective function (Pradella et al., 2013). In the scenario of air pollution, this observation is critical since emphasizes the important interactions between air pollutant concentrations, specific mixtures of pollutants, interactions between inflammatory mediators themselves i.e., G-CSF and IL6 (Yan et al., 2013a) and an expected differential response to cocoa intervention on target neuroprotective cytokines.

One thing is clear, decreasing ET-1 concentrations in urban children using functional foods is a promising avenue (Ghosh and Scheepens, 2009). Given the extensive detrimental effects of ET-1 on the vasculature, the heart, kidneys, the endocrine and nervous systems (Ghosh and Scheepens, 2009; Gonsalves and Kalra, 2010; D'Haeseller et al., 2011, 2013; Kohan et al., 2011; Jacobs et al., 2013; Kaoukis et al., 2013; Kolettis et al., 2013; Mauricio et al., 2013; Nacci et al., 2013; Tarantini et al., 2013; Yuen et al., 2013) the cocoa intervention is warranted.

The effects of cocoa and dark chocolate include improvement of endothelial function, marked reduction in oxidative stress and in the production of pro-inflammatory cytokines, eicosanoids, and in platelet activation (Akita et al., 2008; Selmi et al., 2008; Ghosh and Scheepens, 2009; Monagas et al., 2009; Panneerselvam et al., 2010; Spadafranca et al., 2010). There are significant neuroprotective effects of cocoa flavonols including angiogenesis, neurogenesis in regions involved in learning and memory, cognition improvement, positive effects on mood, and a significant correlation between chocolate consumption per capita and Nobel laureates (Fisher et al., 2006; Francis et al., 2006; Patel and D'Souza, 2008; Scholey et al., 2010; Field et al., 2011; Desideri et al., 2012; Messerli, 2012; Vauzour, 2012; Williams and Spencer, 2012; Nehlig, 2013). In a meta-analysis review of 1106 subjects examining the short effect of flavonoid-rich cocoa, Shrime et al. (2011) concluded that cocoa consumption significantly improved blood pressure, insulin resistance, lipid profiles and flow-mediated vascular dilatation in adults.

It is worth mentioning that short interventions sometimes fail to support the predicted beneficial effects of flavonols in older adults (Crews et al., 2008), and some studies have shown positive mood states but not cognitive improvement (Crews et al., 2008; Pase et al., 2013), although no such studies have been reported in children. Here, we have reported preliminary evidence of cognitive improvement in children. Although the improvement was marginally significant, still it is impressive that we were able to observe those effects in such a short intervention.

The cocoa, cognition and aging study (Desideri et al., 2012) in elderly subjects is of interest to us because the flavonol consumption improvement in cognitive function was associated with changes in insulin resistance, a factor of great importance with childhood obesity (Tirsi et al., 2013). Tirsi et al. put forward the idea that endothelial dysfunction in childhood obesity may precede cerebro-vascular damage and cognitive impairment in adulthood. This is important, obesity is a problem in Hispanic children and insulin resistance is part of the picture (Moreno et al., 2013; Guerrero-Romero et al., 2013). Thus, cocoa supplementation attenuation of insulin resistance (Grassi et al., 2008; Gu et al., 2013) is a desirable effect in exposed children.

In assessing the potential vascular impact of cocoa in children, is also important to remember that the positive effects are mediated by the absorption of monomeric flavonols in the small intestine, thus the type of carbohydrate content is important (Rodriguez-Mateos et al., 2012) and so is the vehicle to administer the cocoa, i.e., milk, since lactase non-persistance, lactose maldigestion and lactose intolerance could interfere with the cocoa absorption (Heaney, 2013).

A critical issue in exposed children was their response of PMN, and selected cytokines with PM_2.5_ and ozone concentrations. We have previously reported the marked decrease of total neutrophils as evidence of endothelial dysfunction, and the trapping of PMN in the brain capillaries likely contributing to hypoperfusion (Calderón-Garcidueñas et al., 2007). The association between total eosinophils and basophils and PM_2.5_, PM_10_ and ozone is a new finding in our cohorts and likely relates to bone marrow responses as described by Tan et al. (2000) These authors commented about the bone marrow differential peripheral blood and time responses depending on the concentrations of air pollutants and by the fact that peripheral blood counts could also be influenced by exercise and stress (Tan et al., 2000). The significant differences in WBC turnover and transit times in peripheral blood, diurnal and ethnic variations in CBC values, and blood sampling time could also account for the differential responses related to air pollutant cumulative values.

Equally interesting, in this study MRS ratio key variables: N-acetylaspartate/creatine (NAA/Cr), choline/creatine (Cho/Cr), and myoinositol/creatine (mI/Cr) in the prefrontal white matter, but not in hippocampus, were associated with cumulative PM and ozone concentrations. These findings are relevant in the context of air pollution exposures because these ratios are particularly useful for assessing neuroinflammatory, dementing and vascular disorders and the concentrations of these metabolites typically correspond to disease severity and often correlate well with clinical variables including cognition (Watanabe et al., 2012; Shiino et al., 2012; Chang et al., 2013; Saito et al., 2013). In patients with mild cognitive impairment and Alzheimer's disease, negative correlations between left hippocampal myoinositol (MI) and verbal memory, general memory and delayed recall and the right hippocampus and verbal memory are seen (Watanabe et al., 2012). Neuroinflammation with activated astrocytes and microglia are often associated with elevated MI while neuronal injury is indicated by lower than normal levels of NAA (Chang et al., 2013). Although, our neuropathological and clinical studies (Calderón-Garcidueñas et al., 2008a,b, 2012a,b, 2013) have shown the brunt of the pathology in the frontal and parietal white matter, the vascular and inflammatory changes are diffuse. Thus, the finding of frontal white matter metabolite ratios associated with air pollutant concentrations is not an unexpected finding. Moreover, hemispheric white matter has the lower vascularity v gray matter (Artzi et al., 2013), making the combination of high ET-1 concentrations, capillary trapping of PMN and arteriolar pathology (Calderón-Garcidueñas et al., 2008a,b, 2009, 2012b) potentially critical for metabolic white matter changes associated with air pollutant concentrations. We fully expect to see robust MRS differences in longer intervention studies.

## Looking forward and limitations

Despite controversy regarding the mechanistic pathways involved in the CNS damage associated with exposure to air pollutants, specifically fine and ultrafine particles of diverse origin, we have greatly improved our understanding of the mechanistic processes (Nguyen et al., 2002; Fassbender et al., 2004; Block and Calderón-Garcidueñas, 2009; Krishnan et al., 2012; Nehlig, 2013). Oxidative stress, endothelial dysfunction and neuroinflammation are at the core of the air pollutant brain effects, and the same factors play a key role in neurodegenerative diseases (Evans et al., 2002; Simard and Rivest, 2004; Qin et al., 2007; Campbell et al., 2009; Rivest, 2009; Levesque et al., 2011a,b; Win-Shwe and Fujimaki, 2011; Wu et al., 2011; Castellani and Perry, 2012; De la Torre, 2012; Ho et al., 2012; Lucchini et al., 2012; Nunomura et al., 2012; Rodrigues et al., 2012; Sharma and Sharma, 2012; Trickler et al., 2012; Villeneuve et al., 2012; Calderón-Garcidueñas et al., 2013). We are looking forward to bridging the gap between neuroinflammation and neurodegeneration, and the effects of neuroprotection in the early stages of the exposure process, an issue of importance in childhood and adolescence when cognitive performance is critical (Nehlig, 2013). There is a need for looking into the cocoa flavonol responses in diverse populations residing in megacities across the globe. This is critical, given that detrimental responses to air pollutants depend not only on the components of air pollution and concentrations, but also on the genetic background of the exposed populations, and on a large list of environmental components, including dietary risk factors, educational attainment, etc. (Stern, 2012).

Our pilot results are potentially limited by the characteristics of the air pollutants in MCMA, and the small population selected. Nevertheless, the robust decrease in ET-1 warrants extensive investigations in exposed children in Mexico City and around the world.

## Summary

MCMA children exhibit an early brain imbalance in genes involved in oxidative stress, inflammation, innate and adaptive immune responses, cell proliferation and apoptosis (Calderón-Garcidueñas et al., 2012a). Neuroinflammation, endothelial activation, endothelial cell hyperplasia, the attachment of white blood cells to the endothelial damaged walls with the reduction of the lumen vessel, high plasmatic concentrations of endothelin-1, and the breakdown of the BBB clearly contribute to cognitive impairment and the pathogenesis of neurodegenerative states (Jian et al., 2012; Roher et al., 2012).

Data from our laboratory suggests we might have a 50 year window of opportunity between the pediatric air pollution associated- brain changes, and the time when the patient will show up at the physician's door with mild cognitive impairment or dementia.

The diffuse nature of the neuroinflammation and the neurodegenerative changes observed in exposed children obligates us to agree with Cherniack (2011) that *future studies* (with polyphenols) *should be designed to account for a disease process in which the pathogenic factors may take place for years before disease manifestations take place*, cocoa interventions in urban clinically healthy children with no risk factors for cognitive or neurological deficits qualify for such approach. Facing the current pediatric clinical and pathology evidence in highly exposed children is imperative if we are aiming our efforts to identify and mitigate environmental factors that influence Alzheimer and Parkinson's disease pathogenesis.

There is a severe and woeful deficit of progress in the development of both Alzheimer's and Parkinson's diseases' modifying therapy (Castellani and Perry, 2012; De la Torre, 2012). Since air pollutants likely play a role in the development of neuroinflammation and neurodegeneration, it is very noteworthy, that in the US alone we have >74 million people being exposed to concentrations of PM_2.5_ above the 2006 standards (PM _2.5_ annual standard of 15 μ g/m^3^). Poor urban minority children are at increased risk of exposure from both outdoor and indoor air pollution and disparities based on SES can be very significant (Adamkiewicz et al., 2011; Miranda et al., 2011; Young et al., 2012).

Mechanisms of action of polyphenols, the endothelial improvement responses documented in the literature and the ET-1 results in our pilot study warrant for future polyphenol strategies in air pollution pediatric neuroprotection and the implementation of early preventive pathways (Castellani and Perry, 2012).

Although the two short-term objectives of this pilot study were accomplished, our ultimate goal is to protect exposed children through multidimensional interventions yielding both impact and reach. In particular, *experimental interventions* (D'Angiulli et al., 2012) could be the main tool to determine the extent of reversibility, and conversely, plasticity or neuroprotection, since with the appropriate intervention exposed children's performance could catch up with that of the minimally exposed counterparts. Since the primary goal would be to identify children at risk, possibly at the largest scale, the first step could be to screen entire schools in different neighborhoods with different air pollutant profiles.

The need for multidisciplinary approach to be applied in neuroprotection ought to include the identification of children and teens at risk from the detrimental brain effects of air pollutants by (1) Using air pollution databases from available monitoring stations to evaluate air pollutant profiles in the selected geographic area. (2) Exposures to traffic-related air pollutants at each child's school and current residence with land use regression (LUR) models that combine a geographic information system (GIS) with ambient passive monitoring in the target area. (3) Measurement of cumulative ambient exposures to fine PM (PM_2.5_). (4) Robust baseline information on the oxidative potential and metal content of PM found in the targeted regions. (5) Cognitive standard screening first and, when applicable, more elaborate neurocognitive/neurophysiological testing in a follow up, which could include EEG/ERPs, BAEPs, MRI, fMRI, and MRS.

In short, interventions addressing air pollution brain effects on children and teens ought to be key public health objectives. Cocoa flavonols hold promise as nutraceuticals with neuroprotective activities in the setting of pediatric air pollution exposures.

### Conflict of interest statement

The authors declare that the research was conducted in the absence of any commercial or financial relationships that could be construed as a potential conflict of interest.
